# JACOB: An Enterprise Framework for Computational Chemistry

**DOI:** 10.1002/jcc.23272

**Published:** 2013-04-03

**Authors:** Mark P Waller, Thomas Dresselhaus, Jack Yang

**Affiliations:** Theoretical Chemistry, Organic Chemistry Institute, Westfälische Wilhelms Universität MünsterCorrenstrasse 40, Münster, 48149, Germany E-mail: m.waller@uni-muenster.de

**Keywords:** computational chemistry, batch, enterprise, framework, workflow

## Abstract

Here, we present just a collection of beans (JACOB): an integrated batch-based framework designed for the rapid development of computational chemistry applications. The framework expedites developer productivity by handling the generic infrastructure tier, and can be easily extended by user-specific scientific code. Paradigms from enterprise software engineering were rigorously applied to create a scalable, testable, secure, and robust framework. A centralized web application is used to configure and control the operation of the framework. The application-programming interface provides a set of generic tools for processing large-scale noninteractive jobs (e.g., systematic studies), or for coordinating systems integration (e.g., complex workflows). The code for the JACOB framework is open sourced and is available at: http://www.wallerlab.org/jacob. © 2013 Wiley Periodicals, Inc.

## Introduction

Modern scientific computing has advanced over recent years, with computational chemists having access to immense computing facilities.^1^ There are two common strategies for addressing scalability issues in computing: vertical scaling which increases the resources (and hence performance) for a given node, or horizontal scaling where additional nodes are added to the system to increase the systems overall performance. Although vertical scaling obeys the well-known Moore's law,^2^* horizontal scaling appears to be directing the future of scientific computing. Distributed computing, which couples a cluster of computers coupled via a network, comes with its own set of challenges: for example, complicated programming models are required to solve issues such as throughput (volume of data transferred across the network) and latency (the time taken for a packet of data to be transferred between two points) that occur with horizontal scaling. Academic projects have been ported to a number of distributed computing environments such as grid computing^3^–^5^ open science grid (OSG),^6^ volunteer computing^7^,^8^ Berkeley Open Infrastructure for Network Computing (BOINC),^9^ infrastructure as a service,^10^ and, most recently, to cloud computing. Scientific computing in these environments poses significant challenges when increasing the size of datasets (scale) or when coupling between different software components (systems integration). To meet these challenges, scientists have created generic workflow packages. A workflow can be used as a general tool to orchestrate a complex task into a series of more simple reusable tasks. The workflow can also be used to manage repetitive tasks that are required for processing large-scale datasets. These general-purpose scientific workflows handle the generic infrastructure requirements and typically offer a wide range of common prewritten tasks. Importantly, these workflow solutions are extensible, meaning that users are able to extend the functionality of the workflow when required for instance by creating a new task. Generic workflow packages such as Kepler,^11^ Knime,^12^,^13^ and Taverna,^14^ whereas Chemshell^15^ (Tcl^16^) and the more recent PyADF^17^ (Python^18^) are more suited to systems integration in the area of quantum chemistry. The WebMo^19^ web server couples a user-friendly web flow (workflow concept adapted to a web context) to a range of computational chemistry packages via interfaces, whereas Gabedit^20^ is a more recent graphical user interface for computational chemistry. Overall, all these solutions are able to dramatically increase the productivity of a scientist once they are familiar with a particular software package.

Enterprise is a term that describes organizations such as corporations and government institutions. Such organizations have special requirements for software regarding scalability, robustness, and security. The robustness requirement comes from the critical nature of these applications, where downtime, for example, may cause significant revenue loss in the financial sector. Security is also a concern based on the sensitive nature of financial transactions, or the data protection needed for government institutions. Therefore, software engineering professionals have met these requirements by constructing so-called “enterprise software” solutions. These applications tend to be data-centric, and design patterns have emerged as being particularly useful guidelines. In enterprise environments, the requirements for integration between uncoupled software components across multiple networks and repositories present a significant set of challenges. Enterprise software engineers have developed central administration interfaces and rely heavily on the messaging design pattern to meet these challenges.^21^ Another feature of enterprise software is their reliance on frameworks. A framework is an abstraction where the software provides generic functionality that can be changed by user code, thus providing application specific software. A framework is therefore a universal and reusable software platform for the efficient development of applications. Different framework infrastructures are available for enterprise software development. Recently, frameworks that use the “inversion of control” (IoC) principle† have gained increasing popularity. The inversions of control concept means that not all the dependencies need to be known at compile time but may be resolved at runtime. The flow of a particular application is dictated by the framework itself, thus making the software highly configurable and extendable. Such extensibility can be achieved by overriding particular methods, or via a plug-in-based approach. In this case, the source code of the framework is not supposed to be modified by end-users.

Here, we aim to combine the domains of science and enterprise to create a platform for performing computational chemistry in modern computing environments. To do so, we have developed JACOB by applying rigorous software engineering techniques to make extensible and testable code. A framework is abstract by the fact that it provides only a partial solution; in the case of JACOB, one still needs to add the scientific code, only then does the framework loose its abstract nature and become part of a complete functioning application. This abstraction gap enables the framework's design to be generically applicable, and because the framework was not constructed for any particular specific problem, no specific implementations are presented here. The reader is actively encouraged to envisage his or her own possible use case scenarios. Applications that have already been built on the JACOB framework are reported elsewhere for the interested reader.^23^,^24^

## Implementation

Object orientated programming (OOP) was developed in the 1960s to improve the modularity and scalability of a code base. In OOP, the class takes center stage, where real-world objects from a particular problem domain are mapped on to a set of classes that contain data fields (variables) and methods (procedures). Object orientation helps to create flexible and more manageable code that is built from many relatively uncoupled classes. The classes in the JACOB framework adhere to the enterprise bean convention^25^ and are either plain old java^26^ objects or their dynamic descendant, plain old groovy^27^ objects (POGOs). Spring^28^ is an application development framework for enterprise Java. The Spring framework is responsible for the creation of the “Spring beans” inside the IoC container. The process of defining these relationships between the Spring beans (such as dependencies) is known as wiring. The wiring in JACOB is configured using a number of different technologies. The infrastructure layer is wired using the traditional Exstensible Markup Language (XML)-based configuration. An annotation-based configuration is used for simple beans. Finally, complicated relationships between beans are programmatically configured inside a series of configuration classes. The dependent elements (such as objects or values) are injected into the destination classes automatically according to the wiring specified at run-time. The design pattern is also known as dependency injection and has the added advantage that it makes code more amenable to unit testing.

JACOB adopts the concept of coding to interfaces throughout to facilitate extensibility. The components used to create a single execution of the JACOB framework are therefore highly interchangeable. Implementations for future application code should be written against the interfaces provided by JACOB's application programming interface (API), when possible. We note that modern integrated development environments (e.g., Spring Tool Suite (STS),^29^ Eclipse,^30^ NetBeans,^31^ IntelliJ IDEA^32^) make this extremely easy as stubs for all required methods are automatically created for the developer when implementing a particular interface. The JACOB framework can also be extended via plugins when a suitable interface does not already exist. Abstract classes are also used wherever possible to reduce the size of the code base, while increasing the productivity of development efforts.‡ Clean and transparent coding practices were applied throughout JACOB for clarity of future developers.^33^ Documentation that describes the dependencies of classes and their methods is automatically generated out of the source code using Groovydoc.^34^ In Java, it is trivial to create java interfaces to other traditional compiled languages such as Fortran, C, or C++, for computationally intensive tasks.

In accord with the domain-driven design pattern, the JACOB framework employs a strict separation of concerns, by employing a number of layers:

- Model: In the domain model pattern, objects that are persisted are represented as a set of domain models. Therefore, the model layer contains a set of objects that hold the current state of an application, that is, the data.
- Service: A service is simply a class (or set of classes) that contains code that performs application specific logic. In a scientific application, this would typically include algorithms. Services can be used to change the state of the models, that is, procedures.
- Data access object (DAO): Data access objects are specifically used for persistence. This means that the persistence layer can be decoupled from the service and data layers.
- AOP: Aspect-orientated programming handles the secondary functionality introduced by “cross-cutting” elements. Cross cutting elements make it difficult to adhere to the pure object orientation paradigm. Aspects are methods that are called before or after the execution of other methods.^35^,^36^ Because aspects are not located in the classes they work on, it is therefore possible to decouple their functionality from where it is needed. Aspects can therefore be centralized, added, removed, or changed without changing other parts of the framework.

**Figure 1:**
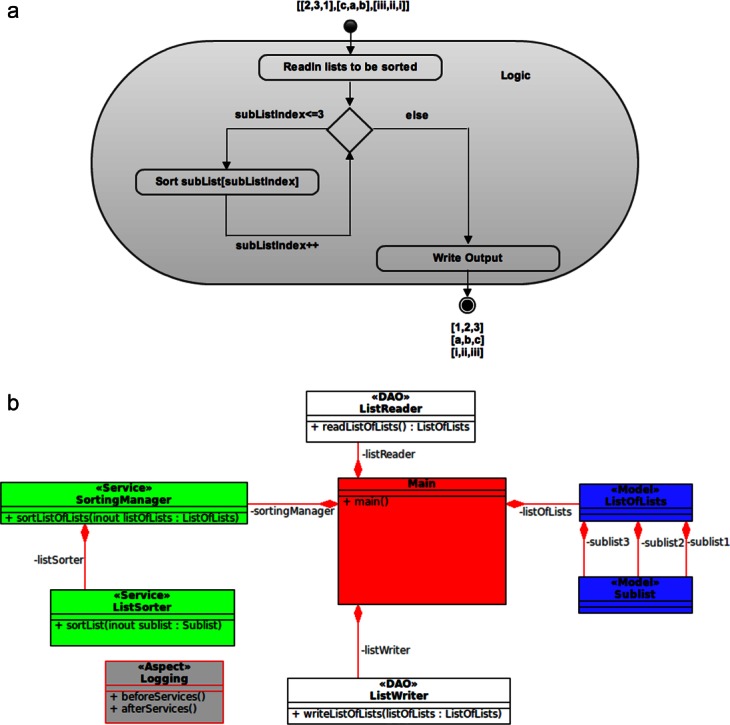
a) an activity diagram and b) domain model diagram [Models (yellow), services (blue), DAOs (green), Aspect (grey), and the Main class (red)] for an illustrative example on how one might map from a workflow to a domain model that is suitable for JACOB.

An illustrative example of the mapping required from a workflow (procedural) to a domain model (object orientated) that is needed for the JACOB framework is shown in Figure 1.

**Figure 2:**
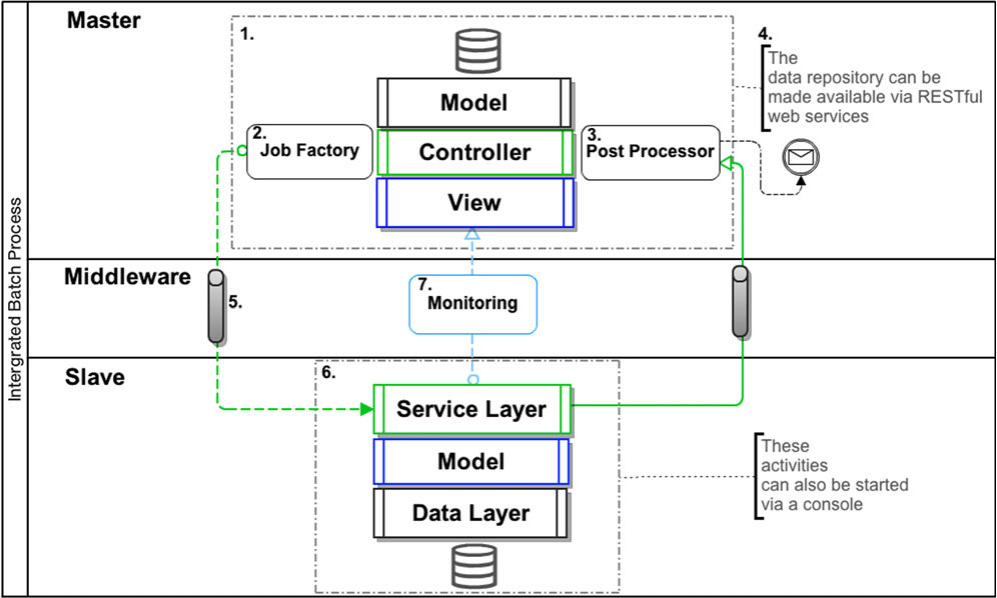
The architecture of the JACOB framework is multilayered and is connected using middleware. [Color figure can be viewed in the online issue, which is available at http://wileyonlinelibrary.com.]

The master–slave architecture was chosen for JACOB due to the nature of computational chemistry where the processing of jobs is typically the bottleneck, and spreading jobs across multiple worker-nodes is often desirable. The master layer is used primarily for user interaction, job setup, interacting with the results, and so on. The slave layer is for computational intensive tasks where the models and services are typically needed to solve scientific problems. There is typically a one-to-many relationship to the slave compute nodes. An overview of the framework infrastructure is given in Figure 2.

Importantly, the ability to select each component, configuring the location of each component, and configuring the communication between the components, is an advantage of using a modular and flexible framework. These options are left to the application developer based on their particular requirements. For instance, if scalability is not an issue, then the slave and master layers of JACOB do not need to be on separate computers. The location of the database holding the results also does not need to be on the master node. The location of the middleware is also configurable.

The configuration of the master and slave layers can be changed using profiles. A profile is a set of user definable configuration options. These profiles can be set depending on the current environment; this efficiently enables multiple “default” configurations of the framework. An environment is an intentionally abstract term that may include production, test, or development. A production environment needs stability, speed, and persistence. On the other hand, in a development environment, it is not convenient to connect to a production database, for example, this may corrupt valuable data, or cause significant delays in the developer time while waiting for the production database to be bootstrapped. The test environment is typically a clone of the production environment, which is useful to reduce the chances of bugs being introduced into production. Only after code is thoroughly tested, for example, performing scientific computation using integration tests, should the production environment be used.

In distributed computing systems, there exists some core systemic requirements namely consistency, availability, and partition tolerance. Consistency meaning that the nodes see the same data, availability means that the system is fully operational, and partition tolerance means that a distributed system can function even when internode communication is severed. Brewer's theorem^37^ states that only two of these requirements can be fully satisfied, and in distributed computing a compromise must be made. Because JACOB is a modular framework, the application developers can make decisions on where to compromise based on their own specific requirements for a given application or environment.

### Master

The web tier of JACOB is developed on the Grails^38^ dynamic web framework that uses the model-view-controller design pattern.

The three main components are:

- Model: the model layer contains a set of objects that hold the current state of an application. A given problem in computational chemistry can be mapped onto an appropriate set of domain models. For example, a job domain model may contain input parameters (e.g., density functional and basis set) and data parsed from input files (e.g., molecular structure). A relational database schema is set up based on the set of domain models. After the job has been processed, the results may be stored in a domain model (e.g., energy and optimized structure) and may be persisted into a database.
- Controller: the controller conveys the message between the model and view. In particular, it converts a map of parameters from user inputs into the model. The controller is also responsible for retrieving data from the model and returns it to the user, for example, as html, XML, or JSON. More computationally demanding tasks can be implemented as services. Services can then be injected into the controllers for post processing, for purposes such as data-mining.
- View: users interact with the framework through the view layer. The view renders a webpage. The webpage can contain a mixture of static html and dynamic content that is retrieved from the model by the controller. Information relating to the current state of the framework can be queried by using search capabilities for selected domain models.

Separating the concerns of the web-application layer reduces the amount of code and its complexity. Typically, an application developer creates the domain models and the services, as these are both domain-specific problems and therefore cannot be handled by a generic framework. The Grails framework then automatically scaffolds a lot of the “boilerplate” code needed for the view and controller layers.

### Job factory web flow

The dynamic web framework automatically handles various repetitive tasks that are prone to errors from manual handling. For example, transferring jobs across multiple nodes, job queuing, job execution, as well as post job data collation. A calculation performed with JACOB will be carried out as following:

- STEP 1: A user may select a specific algorithm for his/her problem from the view page.
- STEP 2: The user can then give the appropriate job parameters for the corresponding algorithm. These parameters will then be persisted into the model layer.
- STEP 3: A HTML5 drag and drop file handler is provided for uploading the input files used for the calculation. A job can only be validated and started if an input file has been uploaded to the application, if appropriate.
- STEP 4: Once a complete job is defined, a message is sent to a message-based middleware broker. A slave job listener (see below) is installed on the compute cluster (node), and is used to receive jobs from the queue. A cluster node will accept, and start a new job in queue (if any), once it becomes free.

### Job post processor

When the calculation is finished, the results will be sent back to the web application and persisted into the database for subsequent analysis or dissemination. A data visualization controller has been implemented for post processing the results of the calculation. The data visualization controller is responsible for retrieving results from the database, performing statistical analysis services, and redirects the analyzed results to the corresponding view page.

**Figure 3:**
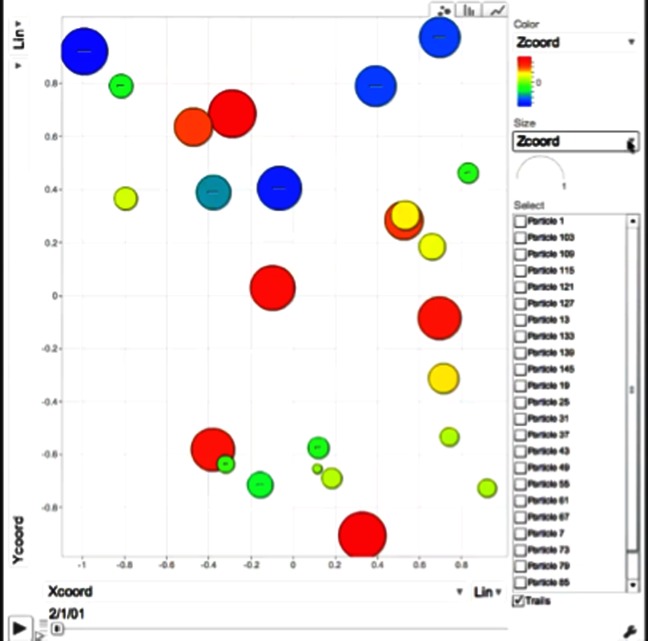
The JACOB framework can render a dynamic graph (Google visualization API) for post processing of job results.

The user can interact with the calculation results in different ways:

1. Browse lists of completed jobs.
2. Perform a query against the completed jobs.
3. Download a log file for the job for further analysis, with the HTML5 file handler.
4. Visualize molecular structure with a Jmol applet.^39^
5. Job results can be visualized using the API from Google's visualization library. An example of such graph is shown in Figure 3.

### Web services

**Figure 4:**
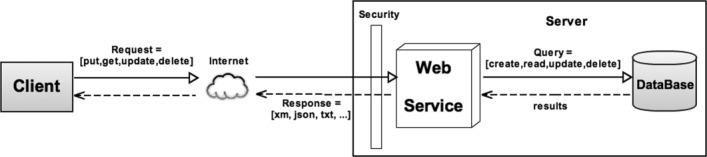
A schematic of the REST web service architecture.

A RESTful web API is used in the JACOB framework to enable data exchange between remote systems. Web services are designed for intermachine communication. REST stands for representational state transfer, and it is an architectural style whereby a client requests a resource from a server using a predefined method. The methods include: GET (retrieves a resource), POST (creates a resource), PUT (updates a resource), and DELETE (deletes the resource). The resource is described by its unified resource identifier. The master layer of JACOB acts as the server and exposes the web services, remote clients then submit a request, and the response is returned from the JACOB server, see Figure 4.

Spring-security^40^-based permissions are used in JACOB to provide a simple mechanism for configuring accessibility to the data repositories. Allowing any user to make a DELETE method call on your scientific results is obviously undesirable. The RESTful web services are language interoperable, and the return format of the message can requested to be in XML, JSON, plain text, and so on.

A user interface is provided via a dynamic web application (Web 2.0), this enables a user to be directed through the application to a specific point via the URL mappings. The end of the URL contains two pieces of information: (a) the domain model from which requested data can be retrieved from or stored into, and (b) a controller action, which specifies the user's intentions with the requested data.

For example:

http://../job/create/

This directs the user to the page where a user can create (controller action) a new job. The corresponding job parameters that the user has entered will be stored in the job domain model, which can be subsequently viewed:

http://../job/show/1

This URL returns a show (controller action) page for job with ID 1 that contains information stored in the run domain model for the corresponding ID.

http://../datavisualization/motionchart?job=2

This directs to a view page that calls the action of plotting a motion chart from the data visualization controller. The data to be plotted should be retrieved from job ID 2.

Therefore, by understanding the URL mappings, and having some knowledge of the data structure, a remote user can interact with the contents of the database via the URLs. However, the view layer can include appropriate hyperlinking, which makes this interaction simpler for the users who do not have interment knowledge of URL mappings or the data structure.

### Message-orientated middleware

The implementation of message-oriented middleware (MOM) such as the apache active MQ^41^ enables loose coupling between the master and slave layers. The master and slave layers therefore do not directly communicate, instead messages are given to a message broker. The messages are stored on the broker until a request is made to process the message. This enables the layers of the systems to communicate asynchronously. Using this paradigm, a form of load balancing is trivially introduced. If the master fails, the responses from the slave nodes will be queued, and only consumed by the master when it is made available again. On the other hand, if the slave(s) fails, the messages will be queued until a slave is again ready to consume the queued requests. The final advantage of this approach is that delivery is guaranteed by using transactions, which increases the robustness of our solution stack.§ The MOM handles the master–slave nature of the client/server mechanism, leaving the more interesting service layer for the future application developers. Of note, a direct connection can be made between the slave nodes and the master if required, for example, via SQL connections.

### Slave

**Figure 5:**
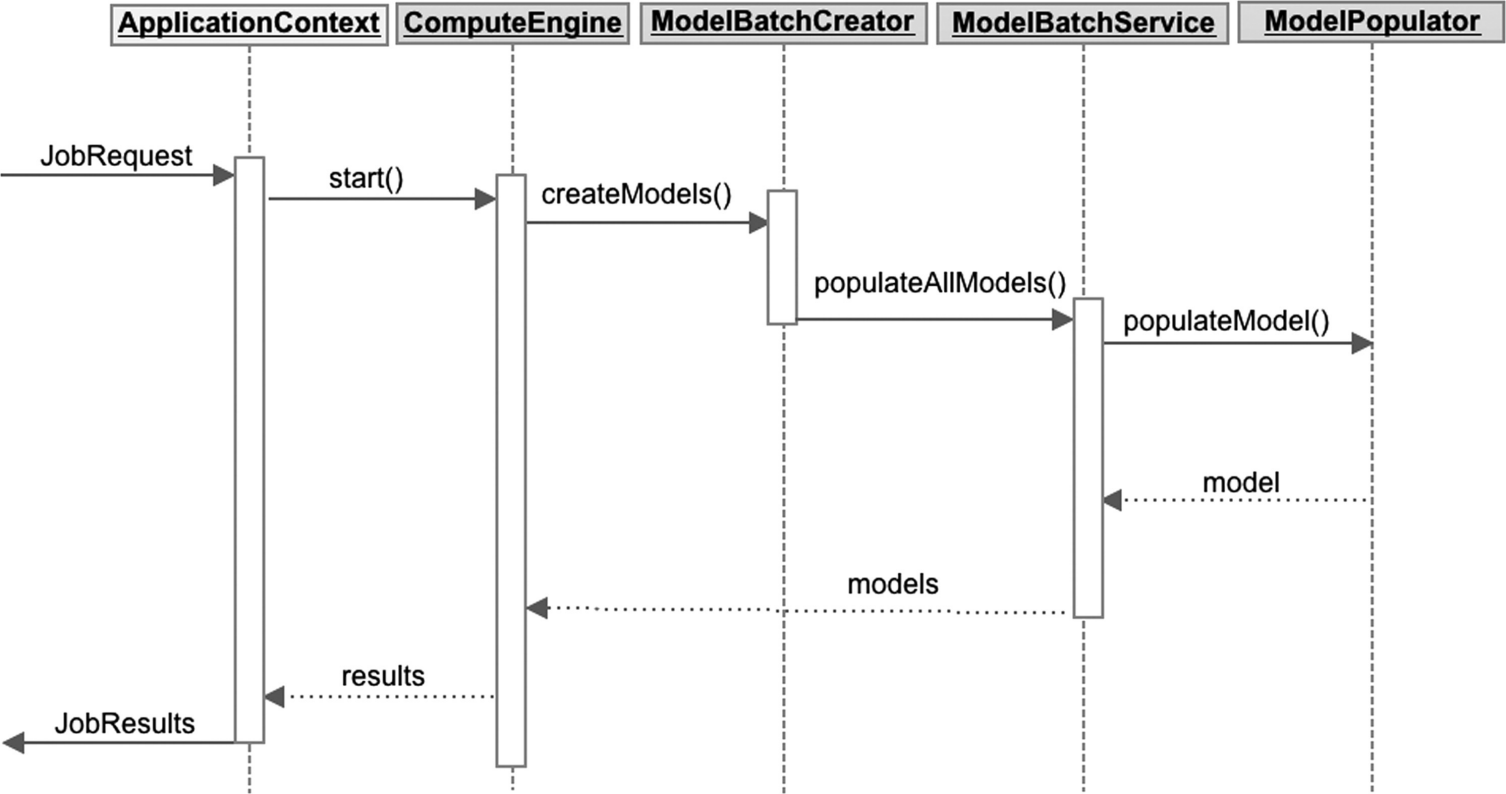
Sequence diagram for the slave tier of the JACOB framework. Interfaces are shown as gray boxes, method calls are on filled arrows, and objects are returned on dotted lines.

The operational flow of the slave layer is given in Figure 5. A *jobRequest* object is received from the MOM, and then the *applicationContext* is built. Only the beans needed for a given job are created in the Spring container. The framework uses a batch-based approach to process a set of models for a specific configuration of the application. We have implemented a sequential (nonthread safe) simple batch service, while for a more robust and efficient implementation, a scalable batch service from Spring batch^42^ partitions the work into “chunks.” The logical flow for a specific computational chemistry job is driven by a *computeEngine*. More specifically, before a job is run, the engine performs preprocessing tasks and calls the batch creator class to generate a set of models. The batch of domain models are then processed on the slave. For example, a set of molecules may be represented by a set of molecule domain models each containing an energy field. The processing of this set includes computing single-point energies and populating the domain models with the parsed energies from the external quantum chemical code wrappers. The *computeEngine* is also capable of post processing the models at the slave level, and then the results are sent back to the centralized master and collated as *jobResults*.

**Figure 6:**
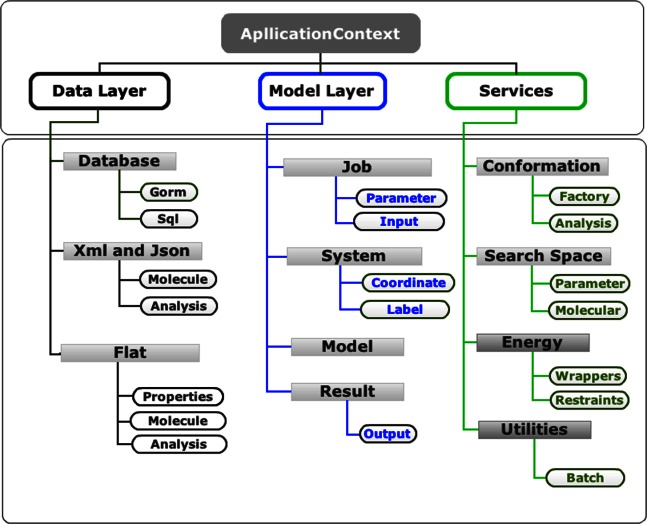
An overview of the important components of the slave tier within the JACOB framework. The application context defines the runtime of the JACOB framework. [Color figure can be viewed in the online issue, which is available at http://wileyonlinelibrary.com.]

The slave component is also separated into layers: this enables a convenient and practical separation of concerns. Some key components are given in Figure 6. More specifically:

- Model layer is used to store the state of objects that are needed for processing a particular job. These set of objects, and their relationships, are defined in POGOs, which are mapped onto a corresponding relational database schema, in the same fashion as on the master layer. For example, a *molecularSystem* is a domain model that stores all of the information that defines the system under investigation, including initial coordinates, atomic labels, and number of molecules.
- Data layer is for persistence within the framework and a diverse range of DAO are available (Fig. 6). An object relational mapping between the domain models and the database is carried out using Gorm^43^ (Hibernate^44^). A traditional structure query language (Sql) DAO is also available for simple create, read, update, and delete operations. Chemical markup language-based XML files can be parsed by the *XmlCoordinateReader* to populate a molecular system. A standard key value pair properties file can be used locally to configure the application, which enables the framework to configure an application in a flexible manner and requires no changes to the code.
- Services constitute the largest component of the framework. A range of generic tools such as conformationFactories for creating conformations and a set of generic structure analysis tools are included in JACOB. Wrappers for a number of quantum chemical programs (e.g., Mopac,^45^ Orca,^46^ Gaussian,^47^ Turbomole^48^) are available and further wrappers are easy to implement. A custom citation annotation is provided for tagging classes that implement algorithms from the scientific literature. A second custom annotation is implemented for tagging classes that wrap external software that details their licensing requirements. Overall, this allows all relevant citations and licenses to be printed out automatically, if, and only if, a particular class had been used in the current configuration of the application.

### Monitoring

The AOP of JACOB handles tasks such as logging^49^ and security,^50^ and monitoring which are examples of secondary functionality that are needed in many different places of an application. Java management extensions (JMX) enables one to modify code (such as adjusting parameters) that is currently running. This is an additional feature that is not normally available for traditional computational chemistry languages such as Fortran or C, where recompilation is required. To make remote modifications to the code, JMX has the ability to monitor the code on-the-fly. This allows data collected from the running application to be used to make informed operational decisions. Due to the nature of batch programming, large datasets can be distributed among many slaves and therefore a monitoring tool is highly desirable.

### Validation

A suite of unit and integration tests have been written to cover a large percentage of the code base using the Spock testing and specification framework.^51^,^52^ Unit tests cover only one particular class, while integration tests are aimed at testing whether multiple classes function together correctly. The test code coverage of the JACOB framework was monitored using the Clover tool.^53^ The Gradle^54^ build automation tool was used to compile, package, manage dependencies, and create the documentation. The set of third party dependencies were automatically synchronized with the Maven central repository.^55^ The Jenkins^56^ continuous integration server to ensure code integrity during development. Continuous integration means the source code was automatically built and the suite of tests were run and logged whenever any code was checked in to the source code repository.^57^,^58^ Performance was monitored using Spring Insight^59^ stress testing framework in a VMware vFabric Tomcat server.^60^ The CodeNarc^61^ framework was used to monitor differences in coding practices among the members of the development team.

## Conclusions

Here, we have developed an integrated framework based on enterprise architecture to create a platform for performing computational chemistry within modern computing environments.

The following design goals were successfully implemented:

- Extensibility: Modern programming concepts were rigorously applied to create an easily extensible framework, leaving future developers more time to solve science-specific problems in a versatile manner.
- Scalability: A master–slave relationship allows dynamic scaling by allocating as many slaves as required for a given job. A centralized master makes monitoring trivial.
- Testability: Continuous integration with a unit and integration test suite ensures code integrity in a team-based development environment.
- Accessibility: User- and role-based authentication and access-control is implemented across the master layer.
- Robustness: The framework is built on top of well-established open source enterprise grade software components.

JACOB is a centralized user-friendly framework for job creation, processing, and analysis that is capable of handling large and complex datasets. Data repositories can be accessed using Web 2.0 technologies or RESTful web services. Therefore, the integrated enterprise framework can be used as an effective collaborative tool for alleviating organizational complexity (e.g., in widespread collaborative research, virtual laboratories, and chemical or pharmaceutical industries). Possible applications of JACOB include systematic studies and/or complicated workflows, for example, in benchmarking, parameter searching, conformational searching, multiscale modelling, docking, or high throughput virtual screening.

## Footnotes

* The lesser known Wirth's law that states “software is getting slower more rapidly than hardware becomes faster.”

† The inversion of control is often described colloquially as the “Hollywood principle” which has arisen from the “Don't call us, we'll call you” phrase. This is a form of implicit invocation.

‡ Adhering to the “Don't Repeat Yourself” (DRY) principle of software design.

§ A “solution stack” refers to the complete set of software components that are used to fulfill the requirements of the system.
